# 1-Isopropenyl-1*H*-1,3-benzimidazol-2(3*H*)-one

**DOI:** 10.1107/S1600536810017897

**Published:** 2010-05-22

**Authors:** Asmaa Saber, Hafid Zouihri, El Mokhtar Essassi, Seik Weng Ng

**Affiliations:** a Laboratoire de Chimie Organique Hétérocyclique, Pôle de Compétences Pharmacochimie, Université Mohammed V-Agdal, BP 1014 Avenue Ibn Batout, Rabat, Morocco; b CNRST Division UATRS, Angle Allal Fassi/FAR, BP 8027 Hay Riad, Rabat, Morocco; c Department of Chemistry, University of Malaya, 50603 Kuala Lumpur, Malaysia

## Abstract

In the title *N*-substituted benzimidazol-2-one, C_10_H_10_N_2_O, the fused ring system is almost planar (r.m.s. deviation = 0.01 Å) and aligned at 57.9 (1)° with respect to the propenyl fragment. In the crystal, adjacent mol­ecules are linked by pairs of N—H⋯O hydrogen bonds into inversion dimers.

## Related literature

For the transformation of 1-isopropenyl-1,3-benzimidazol-2-one to other biologically-active compounds, see: Lakhrissi *et al.* (2010 ▶); Li *et al.* (2010 ▶). A shorter heating time in the synthesis leads to the formation of 4-methyl-2,3-dihydro-1*H*-1,5-benzodiazepin-2-one; see: Saber *et al.* (2010 ▶).
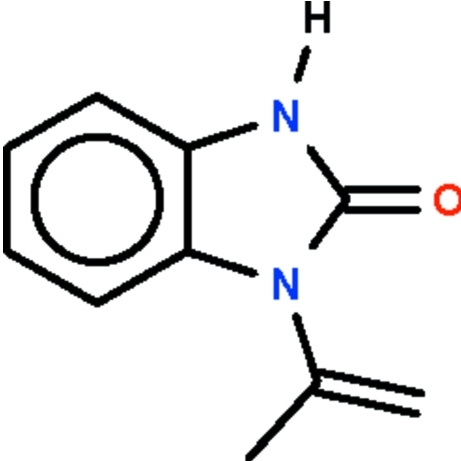


## Experimental

### 

#### Crystal data

C_10_H_10_N_2_O
*M* *_r_* = 174.20
Monoclinic, 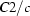

*a* = 15.8724 (2) Å
*b* = 6.0971 (1) Å
*c* = 17.9313 (3) Å
β = 90.961 (2)°
*V* = 1735.07 (5) Å^3^
*Z* = 8
Mo *K*α radiation
μ = 0.09 mm^−1^
*T* = 100 K
0.35 × 0.30 × 0.18 mm

#### Data collection

Bruker X8 APEXII diffractometer
13930 measured reflections
2506 independent reflections
2231 reflections with *I* > 2σ(*I*)
*R*_int_ = 0.023

#### Refinement

*R*[*F* ^2^ > 2σ(*F* ^2^)] = 0.039
*wR*(*F* ^2^) = 0.114
*S* = 0.98
2506 reflections
123 parameters
1 restraint
H atoms treated by a mixture of independent and constrained refinement
Δρ_max_ = 0.39 e Å^−3^
Δρ_min_ = −0.21 e Å^−3^

### 

Data collection: *APEX2* (Bruker, 2008 ▶); cell refinement: *SAINT* (Bruker, 2008 ▶); data reduction: *SAINT*; program(s) used to solve structure: *SHELXS97* (Sheldrick, 2008 ▶); program(s) used to refine structure: *SHELXL97* (Sheldrick, 2008 ▶); molecular graphics: *X-SEED* (Barbour, 2001 ▶); software used to prepare material for publication: *publCIF* (Westrip, 2010 ▶).

## Supplementary Material

Crystal structure: contains datablocks global, I. DOI: 10.1107/S1600536810017897/nc2184sup1.cif

Structure factors: contains datablocks I. DOI: 10.1107/S1600536810017897/nc2184Isup2.hkl

Additional supplementary materials:  crystallographic information; 3D view; checkCIF report

## Figures and Tables

**Table 1:** Hydrogen-bond geometry (Å, °)

*D*—H⋯*A*	*D*—H	H⋯*A*	*D*⋯*A*	*D*—H⋯*A*
N1—H1⋯O1^i^	0.87 (1)	1.95 (1)	2.811 (1)	172 (2)

Symmetry code: (i) 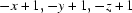.

**Fig. 1.**
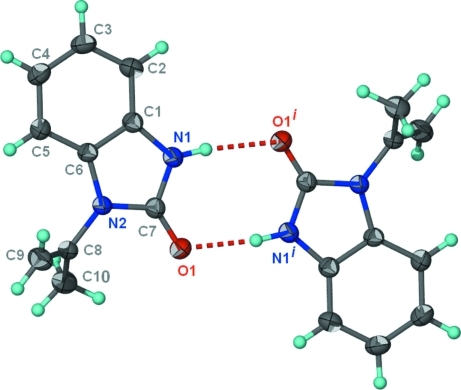
Thermal ellipsoid plot (Barbour, 2001) of the molecule of C10H10N2O at the 70% probability level shown as a hydrogen-bonded dimer; hydrogen atoms are drawn as spheres of arbitrary radius. Symmetry code: i = 1 - x, 1 - y, 1 - z.

## supplementary crystallographic information

### Comment

Benzimidazol-2-one derivatives possess a range of biological and pharmacological activities. Among the many compounds is *N*-isopropenyl benzimidazol-2-one, which can be further converted to 1-acyl-3-isopropenyl benzimidazol-2-ones that are active against *Botrytis cinerea* fungi that affect vegetables and fruits (Li *et al.* 2010). The reagent is also commercially available. We have recently reported the use of this reagent in the synthesis of some glucose-substituted benzimidazol-2-ones (Lakhrissi *et al.*, 2010). For the purpose of understanding the chemistry of these compounds, the crystal structure of the reagent is determined in the present study.

In the molecule of C_10_H_10_N_2_O (Scheme I, Fig. 1), the fused-ring is planar (r.m.s. deviation 0.01 Å); the propenyl fragment is aligned at 57.9 (1) ° with respect to the fused-ring. Adjacent molecules are linked about a center-of-inversion by an N–H···O hydrogen bond.

### Experimental

*o*-Phenylenediamine (1.0 g, 9 mmol) and ethyl acetoacetate (1.2 ml, 9 mmol) were heated in xylene (10 ml) for 6 hours. The mixture was set aside for the growth of colorless crystals of *N*-isopropenyl benzimidazol-2-one; yield 90%. When the heating time is shortened to 1 hour, the product is 4-methyl-2,3-dihydro-1*H*-1,5-benzodiazepin-2-one; details are given in another report (Saber *et al.*, 2010).

### Refinement

Carbon-bound H-atoms were placed in calculated positions (C—H 0.95–0.98 Å) and were included in the refinement in the riding model approximation, with *U*(H) set to 1.2–1.5*U*_eq_(C).

The amino H-atom was located in a difference Fourier map; the N–H distance was restrained to 0.86±0.01 Å. T; the temperature factor of the amino hydrogen atom was freely refined.

### Crystal data

**Table:**

C_10_H_10_N_2_O	*F*(000) = 736
*M**_r_* = 174.20	*D*_x_ = 1.334 Mg m^−^^3^
Monoclinic, *C*2/*c*	Mo *K*α radiation, λ = 0.71073 Å
Hall symbol: -C 2yc	Cell parameters from 8296 reflections
*a* = 15.8724 (2) Å	θ = 2.3–35.0°
*b* = 6.0971 (1) Å	µ = 0.09 mm^−^^1^
*c* = 17.9313 (3) Å	*T* = 100 K
β = 90.961 (2)°	Block, colorless
*V* = 1735.07 (5) Å^3^	0.35 × 0.30 × 0.18 mm
*Z* = 8	

### Data collection

**Table:**

Bruker X8 APEXII diffractometer	2231 reflections with *I* > 2σ(*I*)
Radiation source: fine-focus sealed tube	*R*_int_ = 0.023
graphite	θ_max_ = 30.0°, θ_min_ = 3.4°
φ and ω scans	*h* = −21→21
13930 measured reflections	*k* = −8→8
2506 independent reflections	*l* = −25→25

### Refinement

**Table:**

Refinement on *F*^2^	Primary atom site location: structure-invariant direct methods
Least-squares matrix: full	Secondary atom site location: difference Fourier map
*R*[*F*^2^ > 2σ(*F*^2^)] = 0.039	Hydrogen site location: inferred from neighbouring sites
*wR*(*F*^2^) = 0.114	H atoms treated by a mixture of independent and constrained refinement
*S* = 0.98	*w* = 1/[σ^2^(*F*_o_^2^) + (0.0716*P*)^2^ + 0.9009*P*] where *P* = (*F*_o_^2^ + 2*F*_c_^2^)/3
2506 reflections	(Δ/σ)_max_ = 0.001
123 parameters	Δρ_max_ = 0.39 e Å^−^^3^
1 restraint	Δρ_min_ = −0.21 e Å^−^^3^

### Fractional atomic coordinates and isotropic or equivalent isotropic displacement parameters (Å^2^)

**Table:**

	*x*	*y*	*z*	*U*_iso_*/*U*_eq_	
O1	0.58630 (4)	0.41270 (11)	0.43646 (4)	0.02815 (17)	
N1	0.45280 (5)	0.26700 (13)	0.45678 (4)	0.02451 (18)	
H1	0.4355 (10)	0.366 (2)	0.4880 (7)	0.048 (4)*	
N2	0.53743 (5)	0.08911 (12)	0.38109 (4)	0.02211 (17)	
C1	0.40921 (5)	0.08279 (14)	0.43226 (5)	0.02174 (18)	
C2	0.32851 (6)	0.00950 (17)	0.44634 (5)	0.0275 (2)	
H2	0.2922	0.0883	0.4783	0.033*	
C3	0.30248 (6)	−0.18432 (17)	0.41176 (6)	0.0296 (2)	
H3	0.2473	−0.2386	0.4201	0.036*	
C4	0.35602 (6)	−0.29936 (17)	0.36524 (6)	0.0296 (2)	
H4	0.3367	−0.4314	0.3426	0.035*	
C5	0.43747 (6)	−0.22577 (15)	0.35092 (5)	0.0259 (2)	
H5	0.4740	−0.3051	0.3193	0.031*	
C6	0.46266 (5)	−0.03240 (14)	0.38485 (4)	0.02048 (18)	
C7	0.53105 (6)	0.27304 (14)	0.42624 (5)	0.02226 (18)	
C8	0.60775 (5)	0.04385 (15)	0.33453 (5)	0.02371 (19)	
C9	0.64627 (6)	−0.14799 (17)	0.33983 (6)	0.0338 (2)	
H9A	0.6272	−0.2545	0.3743	0.041*	
H9B	0.6930	−0.1793	0.3091	0.041*	
C10	0.62872 (7)	0.21916 (17)	0.27950 (6)	0.0327 (2)	
H10A	0.6743	0.1682	0.2476	0.049*	
H10B	0.6469	0.3517	0.3062	0.049*	
H10C	0.5788	0.2524	0.2487	0.049*	

### Atomic displacement parameters (Å^2^)

**Table:**

	*U*^11^	*U*^22^	*U*^33^	*U*^12^	*U*^13^	*U*^23^
O1	0.0268 (3)	0.0240 (3)	0.0338 (4)	−0.0062 (2)	0.0053 (3)	−0.0065 (3)
N1	0.0231 (4)	0.0223 (4)	0.0284 (4)	−0.0013 (3)	0.0057 (3)	−0.0049 (3)
N2	0.0214 (4)	0.0209 (3)	0.0242 (3)	−0.0038 (3)	0.0054 (3)	−0.0036 (3)
C1	0.0218 (4)	0.0219 (4)	0.0216 (4)	−0.0003 (3)	0.0012 (3)	0.0005 (3)
C2	0.0215 (4)	0.0315 (5)	0.0298 (4)	−0.0009 (3)	0.0044 (3)	−0.0010 (3)
C3	0.0222 (4)	0.0350 (5)	0.0316 (5)	−0.0075 (3)	0.0017 (3)	0.0001 (4)
C4	0.0282 (5)	0.0304 (5)	0.0302 (4)	−0.0090 (4)	0.0004 (3)	−0.0036 (4)
C5	0.0265 (4)	0.0258 (4)	0.0255 (4)	−0.0044 (3)	0.0034 (3)	−0.0044 (3)
C6	0.0199 (4)	0.0216 (4)	0.0200 (4)	−0.0022 (3)	0.0016 (3)	0.0008 (3)
C7	0.0234 (4)	0.0204 (4)	0.0230 (4)	−0.0008 (3)	0.0027 (3)	−0.0013 (3)
C8	0.0215 (4)	0.0263 (4)	0.0235 (4)	−0.0056 (3)	0.0057 (3)	−0.0045 (3)
C9	0.0292 (5)	0.0292 (5)	0.0433 (6)	−0.0002 (4)	0.0114 (4)	−0.0055 (4)
C10	0.0352 (5)	0.0346 (5)	0.0287 (5)	−0.0088 (4)	0.0090 (4)	0.0014 (4)

### Geometric parameters (Å, °)

**Table:**

O1—C7	1.2338 (11)	C3—H3	0.9500
N1—C7	1.3663 (11)	C4—C5	1.3963 (13)
N1—C1	1.3868 (11)	C4—H4	0.9500
N1—H1	0.871 (9)	C5—C6	1.3827 (12)
N2—C7	1.3878 (11)	C5—H5	0.9500
N2—C6	1.4016 (10)	C8—C9	1.3225 (14)
N2—C8	1.4319 (11)	C8—C10	1.4960 (13)
C1—C2	1.3839 (12)	C9—H9A	0.9500
C1—C6	1.3999 (11)	C9—H9B	0.9500
C2—C3	1.3939 (14)	C10—H10A	0.9800
C2—H2	0.9500	C10—H10B	0.9800
C3—C4	1.3907 (14)	C10—H10C	0.9800
			
C7—N1—C1	110.29 (7)	C4—C5—H5	121.5
C7—N1—H1	122.5 (11)	C5—C6—C1	121.42 (8)
C1—N1—H1	127.2 (11)	C5—C6—N2	131.94 (8)
C7—N2—C6	109.21 (7)	C1—C6—N2	106.63 (7)
C7—N2—C8	124.13 (7)	O1—C7—N1	127.42 (8)
C6—N2—C8	126.52 (7)	O1—C7—N2	125.86 (8)
C2—C1—N1	131.46 (8)	N1—C7—N2	106.72 (7)
C2—C1—C6	121.39 (8)	C9—C8—N2	119.50 (8)
N1—C1—C6	107.14 (7)	C9—C8—C10	124.87 (9)
C1—C2—C3	117.45 (9)	N2—C8—C10	115.54 (8)
C1—C2—H2	121.3	C8—C9—H9A	120.0
C3—C2—H2	121.3	C8—C9—H9B	120.0
C4—C3—C2	120.99 (9)	H9A—C9—H9B	120.0
C4—C3—H3	119.5	C8—C10—H10A	109.5
C2—C3—H3	119.5	C8—C10—H10B	109.5
C3—C4—C5	121.69 (9)	H10A—C10—H10B	109.5
C3—C4—H4	119.2	C8—C10—H10C	109.5
C5—C4—H4	119.2	H10A—C10—H10C	109.5
C6—C5—C4	117.05 (9)	H10B—C10—H10C	109.5
C6—C5—H5	121.5		
			
C7—N1—C1—C2	−178.46 (10)	C8—N2—C6—C5	4.24 (15)
C7—N1—C1—C6	0.49 (10)	C7—N2—C6—C1	1.11 (10)
N1—C1—C2—C3	179.14 (9)	C8—N2—C6—C1	−174.64 (8)
C6—C1—C2—C3	0.31 (14)	C1—N1—C7—O1	−179.45 (9)
C1—C2—C3—C4	0.29 (15)	C1—N1—C7—N2	0.19 (10)
C2—C3—C4—C5	−0.32 (16)	C6—N2—C7—O1	178.83 (9)
C3—C4—C5—C6	−0.25 (15)	C8—N2—C7—O1	−5.29 (15)
C4—C5—C6—C1	0.85 (13)	C6—N2—C7—N1	−0.81 (10)
C4—C5—C6—N2	−177.89 (9)	C8—N2—C7—N1	175.06 (8)
C2—C1—C6—C5	−0.91 (13)	C7—N2—C8—C9	127.09 (10)
N1—C1—C6—C5	−179.99 (8)	C6—N2—C8—C9	−57.76 (13)
C2—C1—C6—N2	178.11 (8)	C7—N2—C8—C10	−56.22 (12)
N1—C1—C6—N2	−0.97 (9)	C6—N2—C8—C10	118.93 (9)
C7—N2—C6—C5	179.99 (9)		

### Hydrogen-bond geometry (Å, °)

**Table:**

*D*—H···*A*	*D*—H	H···*A*	*D*···*A*	*D*—H···*A*
N1—H1···O1^i^	0.87 (1)	1.95 (1)	2.811 (1)	172 (2)

Symmetry codes: (i) −*x*+1, −*y*+1, −*z*+1.
